# Immunity to Influenza Viruses and Vaccines: From Broader Immunity to Chrono-Optimization and Safety

**DOI:** 10.3390/vaccines14060527

**Published:** 2026-06-14

**Authors:** Rongbao Gao

**Affiliations:** NHC Key Laboratory of Biosafety, NHC Key Laboratory of Medical Virology and Viral Diseases, National Institute for Viral Disease Control and Prevention, Chinese Center for Disease Control and Prevention, 100 Changbai RD, Changping District, Beijing 102206, China; gaorb@ivdc.chinacdc.cn; Tel./Fax: +86-1058900854

## 1. Introduction

Influenza viruses remain a formidable challenge to global public health, causing annual epidemics and intermittent pandemics, and resulting in over 3 million severe infections with 290,000–650,000 deaths annually [1,2]. It also frequently causes large-scale outbreaks that spread across countries or continents, and global warming and extreme weather events have intensified the spread and impact of the disease [3]. While vaccination is the cornerstone of control on influenza, the continuous antigenic drift and shift in viruses relentlessly outpace traditional vaccine strategies. The collection of articles presented in this Special Issue provides a compelling snapshot of the current state of influenza vaccine research, highlighting a decisive shift from merely inducing neutralizing antibodies towards a more holistic understanding of immunity, host factors, and vaccine deployment. These studies collectively advance the field by exploring conserved epitopes for universal protection, novel routes of administration, the critical role of non-neutralizing functions, the benefits of vaccination beyond infection prevention, the intricate influence of circadian biology on vaccine success, and the global influenza pandemic preparedness based on vaccine development.

## 2. Overview of Contributions to the Special Issue

### 2.1. Epetopoic Targets and Designation Strategy of Influenza Universal Vaccine

Hemagglutinin (HA), composed of two parts of the globular head (HA1) and the rod-like stem (HA2), is the most abundant glycoprotein on the surface of influenza viruses and plays a key role in the process of viral invasion and infection. One of the most significant hurdles in influenza vaccinology is the hypervariability of the HA head, the primary target of traditional strain-specific vaccines. A critical mass of research is now focused on the conserved HA stem domain. In a detailed study by Nishiyama et al., the authors evaluated Fc-mediated antibody responses (ADCC and ADCP) following seasonal vaccination in humans. In their findings, while the vaccine induced primarily head-specific neutralizing antibodies, it also stimulated a significant increase in HA stem-specific IgG1 in 72% of recipients. And these stem-specific antibodies correlated strongly with cross-reactive ADCC and ADCP activity against divergent influenza strains (H5N1, H7N9), even in the absence of cross-neutralization [4]. This work demonstrates that current vaccines, while imperfect, do leverage Fc-mediated functions to provide a degree of cross-protection, albeit likely insufficient for full clinical protection on its own. It supports the ongoing push for universal vaccines explicitly designed to boost these stem-targeted, Fc-dependent responses. Complementing this effort to broaden immunity, Naoko Uno et al. explored a practical, game-changing strategy to repurpose the existing commercial vaccine Fluzone and Flublok for intranasal delivery. The results showed that intranasal administration with novel adjuvants (a STING agonist, CDA, or a TLR4/7/8 agonist, TRAC478) elicited robust systemic and mucosal immune responses in mice. Notably, the adjuvanted vaccines induced high levels of anti-HA IgA in the bronchial lavage fluid and protected against both H1N1 and H3N2 challenge [5]. This research addresses the critical need for a delivery system in development of vaccines. The differential efficacy of the two adjuvants—with TRAC478 showing better performance against H3N2—underscores the fact that the choice of adjuvant is as crucial as the antigen itself for tailoring the immune response.

### 2.2. Vaccine’s Impact Beyond the Respiratory Tract

Several lines of evidence have demonstrated that influenza vaccination may induce nonspecific effects that go beyond protection against infection [6]. In parallel with efforts to improve the vaccine itself, a study by Akhtar et al. examined the vaccine’s impact beyond the respiratory tract. In a large case–control study from Australia’s severe 2017 H3N2 season, they found that the standard-dose quadrivalent influenza vaccine was 85% effective in preventing hospitalization for cardiovascular disease (CVD), despite having low efficacy against influenza infection that year. This finding is remarkable. It suggests that the vaccine’s benefits are not solely reliant on preventing infection. The authors hypothesize that the vaccine may prevent influenza-related complications (e.g., plaque rupture and inflammatory storms) or have broader immunomodulatory (“pleiotropic”) effects that stabilize cardiovascular health [7]. This adds significant weight to the public health argument for widespread vaccination, especially in older adults and those with chronic conditions, where the primary benefit might be the prevention of serious, non-respiratory outcomes.

### 2.3. The Applications of Adjuvant or Cell Culture-Based Platform in Influenza Vaccines

Artiaga et al. provide a crucial cautionary tale while many studies focusing on efficacy, safety and robust preclinical models remain paramount. They tested the potent iNKT cell agonist α-Galactosylceramide (αGC) as an adjuvant for an inactivated influenza vaccine in a swine model. However, rather than enhancing protection, αGC triggered severe Vaccine-Associated Enhanced Respiratory Disease (VAERD) upon heterologous challenge. Unlike conventional oil adjuvants that drive VAERD through non-neutralizing HA2 antibodies, αGC induced a massive infiltration of IFN-γ-secreting T cells into the lungs, leading to immunopathology [8]. This study sounds a clear warning that powerful immunomodulators like αGC must be carefully vetted in relevant large animal models before human use, as the mechanisms of protection and harm can be distinctly different. In addition, a phase 3 trial by Yun et al. evaluated a full 0.5 mL dose of a cell-culture-based quadrivalent inactivated vaccine (NBP607-QIV) in children aged 6–35 months. The study demonstrated non-inferiority to a licensed vaccine for shared strains and met regulatory criteria for the additional B/Yamagata strain, with a comparable safety profile. The use of a cell-culture-based platform is significant, as it avoids egg-adaptive mutations that can reduce vaccine effectiveness [9]. This study provides critical data supporting the use of full-dose quadrivalent vaccines in this vulnerable age group.

### 2.4. The Global Influenza Pandemic Preparedness

The H5N1 avian influenza virus, particularly the clade 2.3.4.4b lineage, is recognized as a high-risk candidate for future pandemics [10]. The need for updated vaccine strains is dramatically illustrated by the ongoing epizootic of H5N1 clade 2.3.4.4b. Nguyen et al. evaluate a novel H5N1 vaccine candidate (rgPR8/VN23) for the clade 2.3.4.4b highly pathogenic avian influenza virus. Using reverse genetics, the authors generated an inactivated oil-adjuvanted vaccine from a representative Vietnamese field strain. In juvenile chickens, a single dose provided strong protection, markedly reduced viral shedding, and conferred clinical immunity as early as 8 days post-vaccination. However, in 40-week-old laying hens, a single dose offered only partial protection, whereas a double-volume regimen improved efficacy. The study underscores the importance of antigenic matching and age-specific vaccination strategies for effective HPAI control [11]. In addition, a review by Medina-Artiles et al. provides a timely analysis of the pandemic potential of this virus, which has shown a remarkable capacity to infect a widening range of mammals, including recent outbreaks in dairy cattle. The authors emphasize that antigenic mismatch between stockpiled vaccines and circulating clade 2.3.4.4b viruses necessitates the selection of new candidate vaccine viruses (CVVs). They identify CVVs derived from A/Astrakhan/3212/2020 (H5N8) as the most suitable antigenic prototype for the current dominant clade [12]. This work is a call to action for global pandemic preparedness, emphasizing the need for continuous surveillance, rapid CVV selection, and the availability of potency reagents to enable swift vaccine production.

### 2.5. Application of Animal Models and the Temporal Rhythm of Vaccination in Host

Two foundational reviews in this Special Issue summarize the complex landscape of animal models and the impact of circadian and sleep factors on influenza vaccine-induced immune responses. One compares the strengths and weaknesses of various influenza animal models to help researchers select the most appropriate system for their specific question, and summarizes the progress on the construction of mouse-adapted influenza viruses to overcome species-specific barriers [13]. Meanwhile, the paper by Wei et al. demonstrates the power of humanized HLA transgenic mouse models to screen for T-cell epitopes, offering a pathway to design vaccines that harness the power of cellular immunity independent of strain-specific antibodies [14]. The other review by Livieratos et al. synthesized the evidence on how vaccination timing and sleep influence immunity [15]. The authors found a consistent signal that morning vaccination (9–11 AM) is associated with higher antibody titers, particularly in older adults. Furthermore, short sleep duration in the nights preceding vaccination was linked to reduced antibody responses. Although the evidence has limitations and effect sizes are modest, this review opens the door to “chrono-immunization” of a low-cost, non-invasive strategy to potentially enhance vaccine efficacy by simply aligning administration with the body’s biological rhythms.

## 3. Conclusions

These ten articles paint a picture of a field in dynamic evolution. We are moving beyond a singular focus on strain-matched neutralizing antibodies to embrace a multi-pronged strategy. This includes leveraging Fc-effector functions against conserved domains (the universal vaccine dream), optimizing delivery routes for mucosal protection, re-evaluating vaccine benefits for cardiovascular health, timing administration for circadian advantage, and ensuring safety through rigorous preclinical models. While challenges like the ever-present threat of a novel pandemic H5N1 strain persist, the insights from this collection highlight the evolving scope of vaccinology, including developing the next generation of more effective, safer, and more broadly protective influenza vaccines.

## Data Availability

No new data were generated in this study.
